# BATTLING HEAT: COMMUNITY ENGAGEMENT AND HEALTH STRATEGIES FOR LOW-INCOME OLDER ADULTS LIVING ALONE

**DOI:** 10.1093/geroni/igae098.4128

**Published:** 2024-12-31

**Authors:** YunYing Zhong, Jianwen Li, Ajani Jackson, Dahee Kim, Ladda Thiamwong, Jennifer Crook, Christopher Emrich, Rui Xie

**Affiliations:** University of Central Florida, Orlando, Florida, United States; City University of Macau, Macau, Macao, Macau; UCF Intrepid Scholar, Orlando, Florida, United States; University of Central Florida, Orlando, Florida, United States; University of Central Florida, Orlando, Florida, United States; University of Central Florida, Orlando, Florida, United States; University of Central Florida, Orlando, Florida, United States; University of Central Florida, Orlando, Florida, United States

## Abstract

Low-income older adults living alone are at higher risk for falls, social isolation, and worsening functions. The impacts of extreme heat on their health are worthy of further investigation. This study investigates the data from 45 low-income older adults living alone (M=76.6 years old, SD =7.42) in Florida to understand their responses and adaptation strategies to extreme heat or hot days. Most respondents (66.7%) reported discomfort, ranging from slightly uncomfortable (24.4%), uncomfortable (13.3%), very uncomfortable (24.4%), to extremely uncomfortable (4.4%). Less than half of participants (42.2%) reported feeling hot or very hot, and 71.1% reported feeling moderately thirsty to very very thirsty. Overall, participants rated the threat of hot weather on health and day-to-day activities as 5.44 out of 10 (SD=3.25). Despite participants recognizing the potential health risks, there is a disconnect between the perceived threat and actions taken to decrease it. Majority of them did not stay inside (64.4%), nor reduce physical activity (75.6%), nor visit a cool place (93.3%), nor stay hydrated (60.0%), nor take cold bath/shower (95.6%). They appeared lack of knowledge of managing heat in multiple ways, with most respondents reporting only one way to manage heat (57.8%). Most respondents are participating in programs in local senior centers (53.3%) and they have future interested in community programs on heat prevention (53.3%). These findings emphasize the need for specific community-based interventions that provide health strategies to adapt to the extreme heat to address the immediate comfort and health concerns of low-income older adults living alone.

